# Medical News and Items

**Published:** 1876-08

**Authors:** 


### Medical News and Items.

PREDETERMINATION OF SEX.—Dr. Preston Capshaw, in the *Phil. Med. and Surg. Rep.*, revamps the old French idea of begetting childen of either sex at will. Stock raisers, it is claimed, by regarding the conditions necessary to this predetermination, are enabled to control the sex of colts and calves. When a *female* product is desired, sexual congress is permitted only at the *earliest* period of the erotic fever. When the *male* young is desired, the sexes are to be kept apart till the climax of erotism is passed. Applying this rule to human beings, the Dr. has found abundant instances to confirm his views. Coition just *prior* to menstruation begets girls; *after* menstruation, boys. Sexual intercourse abstained from for a period of ten or fifteen days *after* menstruation, constitutes a trespass upon the succeeding period, and the result will consequently be a girl. This theory is not a new one. It has been before the profession for many years, and its conspicuous failure in well authenticated cases has been very, very often annotated; so that at the present time it is almost wholly disregarded by physicians. An acquaintance of ours, a very intelligent doctor, who firmly believed the theory, one day expatiated at some length on the certainty of this method of predetermination, and boastingly wound up his remarks by announcing that his residence would furnish, within thirty days, an illustration of the triumph of the theory; it was to be a boy. Within the time specified the doctor's family was increased to the extent of TWO LITTLE GIRLS!!

---

THE SHOWER OF FLESH IN KENTUCKY.—Dr. A. McL. Hamilton says he has examined, with Dr. J. W. I. Arnold, a piece of the substance of this famous shower, sent to Prof. Chandler of New York. He says they found the piece—ten lines in length by four in breadth—to be lung tissue. There were bronchi, blood vessels, air cells, etc.

Dr. L. D. Kastenbine, who has examined specimens of the matter deposited, declares that he found it to be muscular fibre, that it was voluntary muscle and that he saw the striae distinctly. "On heating a small portion on a platinum spatula," over a burner, "it melted and burned with a 'spurting flame,'" "and the odor was distinctly like rancid mutton," and was so pronounced by meat experts.

On the other hand we are told the substance found, and supposed to have been deposited in the shower, was nothing but a fungous vegetable growth that develops very rapidly—develops in a single night—from minute germs carried by the winds, and that therefore there was nothing remarkable about the appearance.

We hope somebody will write the true story of the phenomena of that "shower," and in a way, and attended by an array of facts, and proof of them, that will make the account past gainsaying.

---

A New Stomatoscope.—Dr. Bruck, Jr., a *private docens* of dentistry, in Breslau, has discovered a new mode of illuminating the teeth and cavity of the mouth. The apparatus enables the practitioner to detect the slightest morbid changes in the teeth, and so great is the intensity of the light (to produce which the Drummond lime light is employed) that even the roots of the teeth within the jaw can be recognized. —*Med. Times and Gaz.*, April, 1876.

---

School children's eyes suffer more or less from injured vision, when the school room has windows on both sides; and Germany thinks so seriously of the matter that a law has been passed in that empire, forbidding bilateral windows in schools.—*Times and Gazette.*

---

The way they do Things in France. Attempted Personation at Examinations.—T. Boiron has just been tried at the assizes, with one Coure, an *employé* at the Ville-de-Paris, who had obtained for his accomplice a false certificate, in order to obtain inscription at the Faculty at Montpelier, and enable him to pass the examinations in place of one Crespin, who had fled to Spain. Boiron and Coure were both found guilty, the former being condemned to five years' penal servitude and the latter to five years' imprisonment."—*Gaz. Hebd.* April 7, 1876.—*Times and Gaz.*

NECROLOGICAL.—M. Béhier, Professor of Clinical Medicine, in the Hôtel Dieu, died in Paris, May 8.

DR. LUDOVIC HERSCHFELD, Professor of Anatomy, in the University of Warsaw, and the author of the well known Atlas of Plates of the Nervous System, died from dropsy, on May 10.—*Ex.*

